# Rapid Degradation Pathways of Host Proteins During HCMV Infection Revealed by Quantitative Proteomics

**DOI:** 10.3389/fcimb.2020.578259

**Published:** 2021-01-27

**Authors:** Kai-Min Lin, Katie Nightingale, Lior Soday, Robin Antrobus, Michael P. Weekes

**Affiliations:** Cambridge Institute for Medical Research, University of Cambridge, Cambridge, United Kingdom

**Keywords:** human cytomegalovirus, HCMV, quantitative proteomics, protein degradation, proteasome inhibitors, tandem mass tag (TMT), proteasome, lysosome

## Abstract

Human cytomegalovirus (HCMV) is an important pathogen in immunocompromised individuals and neonates, and a paradigm for viral immune evasion. We previously developed a quantitative proteomic approach that identified 133 proteins degraded during the early phase of HCMV infection, including known and novel antiviral factors. The majority were rescued from degradation by MG132, which is known to inhibit lysosomal cathepsins in addition to the proteasome. Global definition of the precise mechanisms of host protein degradation is important both to improve our understanding of viral biology, and to inform novel antiviral therapeutic strategies. We therefore developed and optimized a multiplexed comparative proteomic analysis using the selective proteasome inhibitor bortezomib in addition to MG132, to provide a global mechanistic view of protein degradation. Of proteins rescued from degradation by MG132, 34–47 proteins were also rescued by bortezomib, suggesting both that the predominant mechanism of protein degradation employed by HCMV is *via* the proteasome, and that alternative pathways for degradation are nevertheless important. Our approach and data will enable improved mechanistic understanding of HCMV and other viruses, and provide a shortlist of candidate restriction factors for further analysis.

## Introduction

Human cytomegalovirus (HCMV) is a ubiquitous betaherpesvirus that persistently infects the majority of the human population worldwide (Cannon et al., 2010). Following primary infection under the control of a healthy immune system, a latent infection is established that persists lifelong (Reeves et al., 2005). Although primary infection is mostly asymptomatic in healthy individuals, HCMV may lead to significant morbidity or mortality in immunocompromised patients, particularly transplant recipients and AIDS patients (Griffiths et al., 2015). Vertical transmission of HCMV is a leading cause of congenital infection, resulting in deafness and intellectual disability in newborns (Manicklal et al., 2013). Existing therapies that either target the viral polymerase or terminase are associated with significant toxicity and/or sporadic resistance (El Helou and Razonable, 2019). The identification and characterization of critical facets of host innate immunity that are targeted for degradation by HCMV proteins thus has important implications for antiviral therapy, since such interactions may be inhibitable by small-molecules, facilitating endogenous inhibition of viral replication (Nathans et al., 2008).

HCMV has been reported to disrupt interferon (IFN) production (Kim et al., 2017; Goodwin et al., 2018), neutralize the IFN response (Le-Trilling and Trilling, 2015; Le-Trilling et al., 2020), inhibit natural killer (NK) cell activation (Patel et al., 2018), and avoid T cell surveillance *via* downregulation of MHC molecules (Jackson et al., 2011). Additionally, diverse effects on other key cellular functions have been observed including on cell cycle regulatory proteins and ubiquitin ligases themselves (Weekes et al., 2014; Clark and Spector, 2015; Koshizuka et al., 2016; Koshizuka et al., 2018). A common final pathway for many host protein targets is proteasomal or lysosomal degradation (Halenius et al., 2015; Le-Trilling and Trilling, 2020). HCMV facilitates viral replication by degrading components of cellular promyelocytic leukemia nuclear bodies (PML-NB) Sp100, MORC3, and DAXX that act as restriction factors (Kim et al., 2011; Tavalai et al., 2011; Schreiner and Wodrich, 2013; Sloan et al., 2016). We previously developed three orthogonal proteomic/transcriptomic screens to quantify protein degradation early during HCMV infection, identifying 133 degraded proteins that were enriched in antiviral restriction factors. The power of this approach was demonstrated by our identification of helicase-like transcription factor (HLTF) as a novel restriction factor that potently inhibited early viral gene expression and was targeted by the HCMV protein UL145 (Nightingale et al., 2018). However, a global approach to identify the mechanism of HCMV-induced protein degradation is lacking. Our previous study employed the broad, non-selective inhibitor MG132, which is known to affect lysosomal cathepsins in addition to the proteasome (Wiertz et al., 1996), and leupeptin which is a naturally occurring protease inhibitor that can inhibit some proteasomal proteases in addition to the lysosome (Nightingale et al., 2018).

In this study, we used the selective proteasome inhibitor bortezomib (Chen et al., 2011) to identify proteins specifically targeted for proteasomal degradation during HCMV infection. This identified that the majority of proteins rescued from degradation by MG132 were also rescued by bortezomib, highlighting the role of viral subversion of the proteasome in immune evasion. Our data additionally provide a shortlist of proteins degraded by the proteasome early during infection that are enriched in known antiviral factors for further investigation.

## Material and Methods

### Cells and Cell Culture

Primary human foetal foreskin fibroblast cells (HFFFs) immortalized with human telomerase (HFFF-TERTs, kindly provided by Dr. Richard Stanton at School of Medicine, Cardiff University) (McSharry et al., 2001) were maintained in Dulbecco’s modified Eagle’s medium (DMEM) supplemented with 10% v/v foetal bovine serum (FBS), 100 U/ml penicillin, and 100 μg/ml streptomycin at 37°C with 5% CO_2_ (DMEM/FBS/PS). HFFF-TERTs have been tested at regular intervals since isolation to confirm that human leukocyte antigen (HLA) and MHC Class I Polypeptide-Related Sequence A (MICA) genotypes, cell morphology, and antibiotic resistance are unchanged.

### Virus and Virus Titration

The recombinant HCMV (RCMV1111) used was derived by transfection of a BAC clone of HCMV strain Merlin, the genome of which is designated the reference HCMV sequence by the National Centre for Biotechnology Information and was sequenced after three passages *in vitro* (Dolan et al., 2004; Stanton et al., 2010). Virus stocks were prepared from HFFF-TERTs as described previously (Nobre et al., 2019). Tissue culture supernatants were kept when a 100% cytopathic effect was observed, and were centrifuged to remove cell debris. Cell-free virus was pelleted from supernatant by centrifugation at 15,000×g for 2 h and then resuspended in fresh DMEM. Residual debris was removed from the resulting virus stocks by centrifugation at 10,000xg for 1 min. Virus titration was achieved by intracellularly staining HCMV IE1/2 in HFFF-TERTs that had been infected with serially diluted HCMV. Cells were harvested 24 h post-infection, fixed in 4% paraformaldehyde, permeabilized with ice-cold methanol, blocked with human TruStain FcX Fc receptor blocking solution (Biolegend) and then subjected to primary (anti-HCMV IE1/2, mouse monoclonal 6F8.2, Millipore) and secondary (anti-mouse IgG conjugated with Alexa Fluor 488, Thermo) antibody incubation. Data was acquired by FACSCalibur (BD biosciences) and analyzed with FlowJo software (BD biosciences). The percentage of infected cells was determined by the percentage of IE1/2 positive cells, which was used to calculate the titre of virus stock.

### Virus Infections and Inhibitors

1x10^6^ HFFF-TERTs were plated in a 25 cm^2^ flask. After 24 h, the medium was changed to DMEM lacking FBS but with 4 μg/ml dexamethasone, as this approach has been shown to improve infection efficiency (Tanaka et al., 1984). After 24 h, the medium was changed to DMEM containing the requisite volume of HCMV strain Merlin stock to achieve MOI 5. Cells were gently rocked (5 rpm) for 2 h, and then the medium was changed to DMEM/FBS/PS. MG132 (Sigma) at 10 μM or bortezomib (Sigma) at a range of concentrations was added to the cell culture 12 h prior to sample collection. Bortezomib was used at final concentrations between 50 nM–2 µM. Inhibitors were dissolved in dissolved in dimethyl sulfoxide (DMSO, Sigma), which was used at the same final concentration in both treated and untreated samples. For 12 hpi experiments, inhibitors were added to the initial viral mixture used for infection, which was replaced with drug-containing fresh DMEM after the 2 h of incubation.

### Quantitative Tandem-Mass-Tag Based Proteomics Analysis and Statistical Analysis

Methods of proteomics analysis were described in our previous publication (Nightingale et al., 2018), and are briefly described here with a detailed description in the supplementary information. Whole cell lysates were digested into peptides with LysC and trypsin, and equal amounts of peptide labelled with 10-plex tandem-mass-tag (TMT) reagents. (Thermo, Cat # 90110). Enriched, labelled peptides were subjected to liquid chromatography coupled with multi-stage mass spectrometry (LC-MS3) prior to quantification of ~2,500 proteins in a single mass spectrometry analysis using an Orbitrap Fusion Lumos (Thermo). To acquire more comprehensive data, TMT-labelled peptide samples were subjected to high pH reversed-phase fractionation (HpRP) to generate 12 combined peptide fractions prior to mass spectrometry. Mass spectra were processed using a SEQUEST-based software pipeline for quantitative proteomics, “MassPike”, through a collaborative arrangement with Professor Steven Gygi’s laboratory at Harvard Medical School. Experiments were performed in one biological replicate. The method of significance A was used to estimate the p-value that each ratio was significantly different to 1 using Perseus version 1.5.1.6 (Cox and Mann, 2008). Values were adjusted for multiple hypothesis testing using the method of Benjamini-Hochberg (Cox and Mann, 2008).

### Immunoblot

Cells were lysed with RIPA buffer (Cell Signaling) containing Complete Protease Inhibitor Cocktail (Roche) and then lysates were sonicated with Bioruptor Pico (Diagenode). Protein concentration was measured by BCA kit (Thermo). Lysates were reduced with 6X Protein Loading Dye (Tris 375 mM pH 6.8, 12% SDS, 30% glycerol, 0.6 M DTT, 0.06% bromophenol blue) for 5 min at 95°C. Thirty μg of protein for each sample was separated by PAGE using 4–15% TGX Precast Protein Gels (Bio-rad), then transferred to PVDF membranes using Trans-Blot Systems (Bio-rad). The following primary antibodies were used: anti-GLG1 (MAB78791, R&D Systems) and anti-GAPDH (MAB374, Millipore). Secondary antibodies were IRDye 680RD goat anti-mouse (925-68070, LI-COR) and IRDye 800CW goat anti-rabbit (925-32211, LI-COR). Fluorescent signals were detected and quantified using a LI-COR Odyssey scanner and Image Studio software (LI-COR).

## Results

### Optimization of Bortezomib Concentration for Experiments in HFFF-TERTs

**Figure 1:**
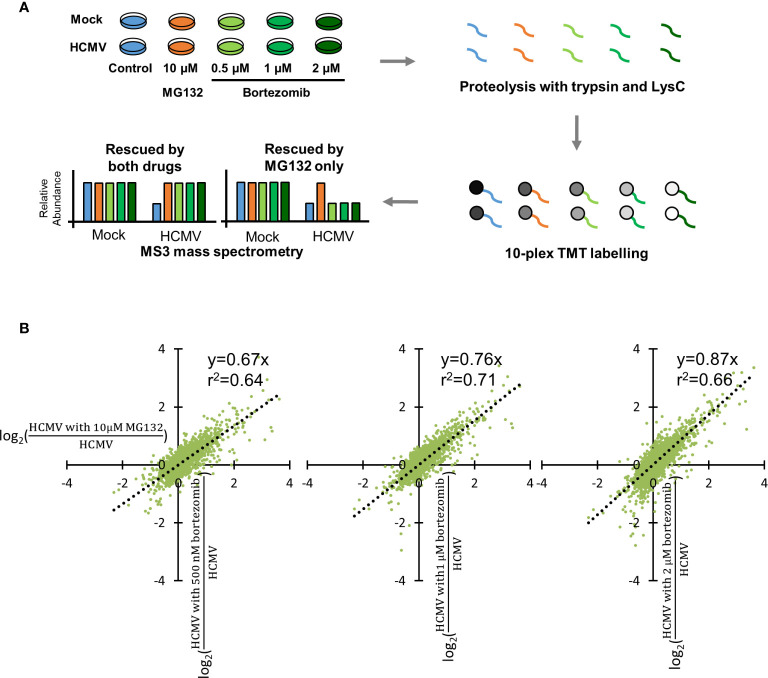
Optimization of bortezomib concentration by comparison with 10 µM MG132. **(A)** Schematic of the experimental workflow. HFFF-TERT cells were infected with Merlin strain HCMV (MOI 5) or mock infected and simultaneously treated with 10 µM MG132, 500 nM, 1 µM, or 2 µM bortezomib. Samples were harvested at 12 hpi to maximize the ability to study very early infection as we previously described (Nightingale et al., 2018). Whole cell lysates were digested into peptides, which were labelled with TMT reagents followed by MS3 mass spectrometry. **(B)** Comparison of 10 µM MG132 with 500 nM, 1 µM, or 2 µM bortezomib during HCMV infection. Each dot represents a protein quantified in the experiment. The x-axis shows the fold change of protein abundance +/- 10 µM MG132 during HCMV infection. The y-axis shows the fold change of protein abundance +/- 500 nM, 1 µM, or 2 µM bortezomib during HCMV infection. The equations and correlation coefficients of the linear trend lines are shown.

Bortezomib has been employed in a number of studies of human cell lines as a specific inhibitor of the proteasome. However, a wide range of concentrations have been used, from 0.1 to 20 µM (Price et al., 2011; Chui et al., 2019). To optimize conditions for proteomic analysis in HCMV-infected immortalized primary human foetal foreskin fibroblasts (HFFF-TERTs), a range of bortezomib concentrations were compared with 10 μM MG132, a concentration we previously showed to provide efficacious inhibition of protein degradation (**Figures 1**, **S1A–B**) (Nightingale et al., 2018). TMT peptide labels and MS3 mass spectrometry enabled very precise protein quantitation, as well as multiplexed analysis of up to 16 samples in the same experiment.

For each protein, ratios of (HCMV with bortezomib)/HCMV and (HCMV with MG132)/HCMV were compared to quantify the relative efficacy of protein rescue. Here, we define “rescue” as the increased expression of a given protein in the presence of inhibitor in the context of viral infection. In order to make an appropriate comparison of fold rescue by both drugs, it was necessary to ensure that a difference could confidently be quantified upon addition of either drug. At lower bortezomib concentrations, rescue ratios were close to 1 with a compressed range of values, making it difficult to assess significance of any given change (**Figure S1B**). The trend of linear correlation and slope of the trend line both increased with increasing bortezomib concentration, with a gradient near to one for 2 µM bortezomib. At this concentration, the degree of rescue was most similar between MG132 and bortezomib, enabling the same fold-change cut off to be applied for both MG132 and bortezomib analyses and 2 µM bortezomib at 12 h post infection (hpi) was therefore selected for detailed assessment (**Figures 1A, B**). A comparison of mock infection in the presence of either inhibitor at optimized concentration identified very similar protein changes (**Figure S1C**), suggesting that although there may be off-target effects of either inhibitor, at a protein level at least these are similar. Furthermore, comparison of protein changes in the presence of MG132 at 12 h of HCMV infection from this and our previous study showed positive correlation, albeit in some cases with different effect sizes on an individual protein level (**Figure S2**, **Table S4**).

### Multiple Host Proteins Are Targeted for Proteasomal Degradation Early During HCMV Infection

**Figure 2:**
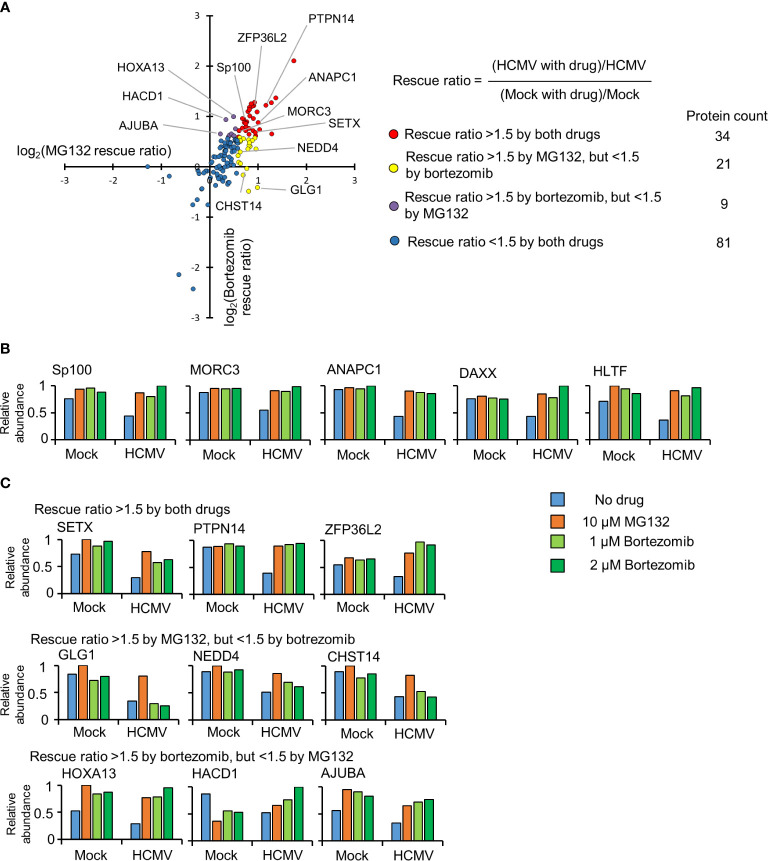
Identification of proteins targeted for degradation by HCMV using an inhibitor-based proteomic screen. **(A)** Results of the inhibitor-based screen. All 145 proteins downregulated >1.5 fold are plotted, with down-regulated proteins divided into 4 groups using rescue ratios of >1.5 as cut-offs. The table shows the number of proteins in each group. For rescue ratios, the denominator (mock with drug)/mock was limited to a minimum of 1 to prevent artificial ratio inflation. **(B)** Examples of positive controls from the existing literature that were validated in this screen. **(C)** Examples of degraded proteins rescued >1.5-fold by both inhibitors (top panels), MG132 but not bortezomib (middle panels), and bortezomib but not MG132 (bottom panels).

To build a comprehensive picture of host protein degradation during the first 12 h of HCMV infection, data from experimental samples described in **Figure 1A** (that included the 2 µM bortezomib condition) was analyzed in detail. Overall, 7,192 host proteins were quantified, 145 of which were down-regulated by HCMV >1.5-fold (with p < 0.01) compared to mock infection. MG132 and bortezomib “rescue ratios” were calculated for each protein, obtained by comparing protein abundance during HCMV infection +/- inhibitor with protein abundance during mock infection +/- inhibitor (**Figure 2A**).

For simplicity and consistency with our previous study (Nightingale et al., 2018), a rescue ratio of >1.5-fold with p<0.01 was set as a threshold to identify proteins rescued by either MG132, bortezomib or both (**Figures 2A**, **Table S1**). Using these criteria, 64/145 (44%) proteins were considered to be rescued by either inhibitor, with 34/64 proteins rescued by both drugs. Notably, this group contained the known HCMV restriction factors Sp100, MORC3, DAXX, and HLTF in addition to cell cycle regulating protein ANAPC1, all of which have been reported to be degraded during HCMV infection by ourselves and others (**Figure 2B**) (Tran et al., 2010a; Chen et al., 2011; Kim et al., 2011; Tavalai et al., 2011; Schreiner and Wodrich, 2013; Sloan et al., 2016; Nightingale et al., 2018).

Data from all proteomic experiments in this study are shown in **Table S2**. Here, the worksheet “Plotter” is interactive, enabling generation of graphs of protein expression of any of the proteins quantified.

**Figure 3:**
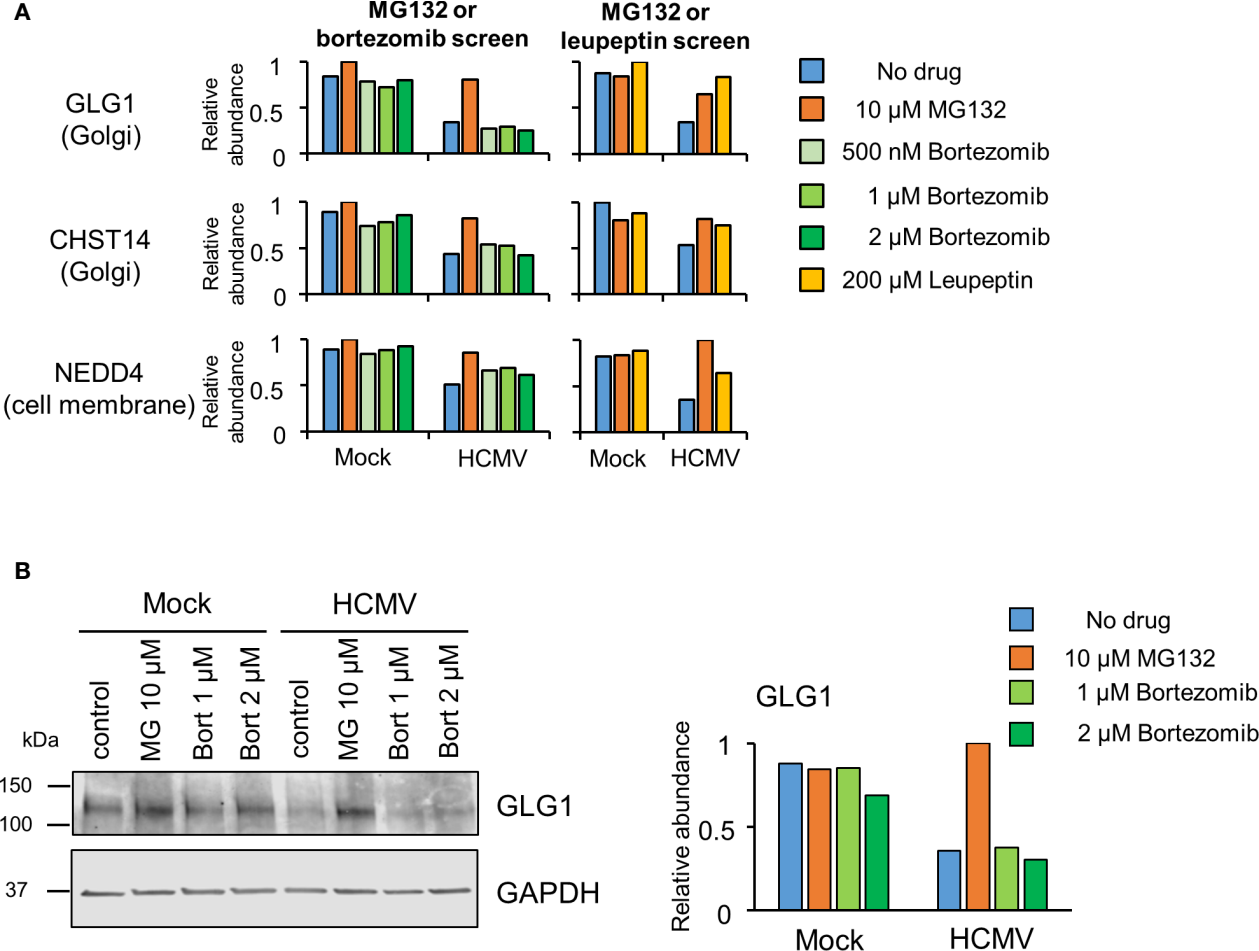
Proteins rescued by MG132 but not bortezomib are also rescued by Leupeptin. **(A)** Results for GLG1, NEDD4 and CHST14, proteins selectively rescued by MG132 but not bortezomib. The left hand panels show data from the complete MG132/bortezomib screen and the right hand panels show the MG132 (10 µM)/Leupeptin (200 µM) screen (12 hpi) described previously (Nightingale et al., 2018). **(B)** (Left panel) Immunoblot showing differential effects of proteasome inhibitors MG132 (MG) and bortezomib (bort) on GLG1 protein during HCMV infection (MOI 5, 12 hpi). (Right panel) Quantitation of GLG1 relative to GAPDH (internal loading control).

Certain proteins exhibited a greater degree of rescue with MG132 compared to bortezomib (**Figure 2B**, yellow dots****). Of the 21 proteins only rescued >1.5 fold by MG132, 13 (62%) exhibited bortezomib rescue ratios of >1.25 and <1.5, suggesting that many of this group of proteins may nevertheless be proteasomally degraded. These included the PDZ domain containing protein 11 (PDZD11) and transcriptional repressor BEN Domain Containing 3 (BEND3) (**Figure S3**, **Tables S1–2**). In contrast, 8/21 proteins appeared genuinely to be selectively rescued by MG132 but not bortezomib (bortezomib rescue ratio <1.25), including the fibroblast growth factor receptor Golgi Glycoprotein 1 (GLG1), E3 ligase NEDD4, and carbohydrate sulfotransferase 14 (CHST14) (**Figure 2C** middle panel,**** **Table S1**). Similar data were obtained for treatments with 500 nM and 1 µM bortezomib, validating these findings, and differential effects of MG132 and bortezomib on GLG1 protein were validated by immunoblot (**Figures 3A, B**). Interestingly, Gene Ontology annotation of all 8 proteins indicated an association with either the cell membrane, the Golgi apparatus, or vesicle secretion. Furthermore, comparison of data with our previous study examining protein rescue by MG132 or leupeptin indicated that GLG1, NEDD4, and CHST14 were also significantly rescued by treatment with the lysosomal protease inhibitor leupeptin (**Figure 3A**), suggesting that a proportion of the proteins rescued by MG132 alone are degraded lysosomally.

Of proteins exhibiting a greater degree of rescue with bortezomib compared to MG132 (**Figure 2A**, purple dots****), 8/9 (89%) exhibited MG132 rescue ratios >1.25 but <1.5 (examples in **Figure S2**, **2C** bottom panel****), suggesting that the majority of all proteins in this class were in fact rescued by both inhibitors. The one exception was LIM domain-containing protein AJUBA, whose MG132 rescue ratio was 1.16 in this data (**Figure 2C** bottom panel****), but neared significance in our previous study (**Table S2**); these differences may reflect relatively poor quantitation by only two or one peptides respectively.

### Proteasomal Regulation of Viral Proteins

**Figure 4:**
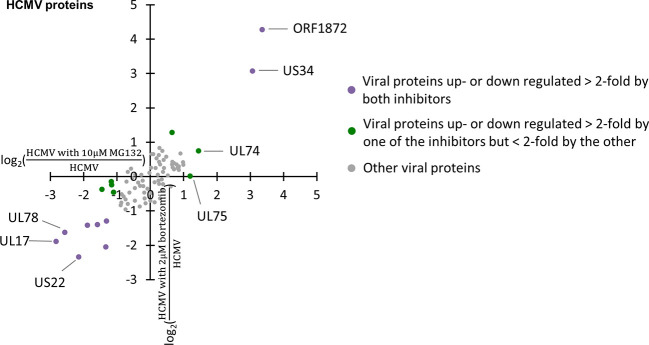
Regulation of 82 viral proteins by proteasome inhibitors. Viral proteins up- or down-regulated >2-fold by both proteasome inhibitors are marked in purple, with proteins up- or down-regulated >2-fold by one proteasome inhibitor, but <2-fold by the other inhibitor marked in green.

The application of MG132 during infection led to significant changes in the abundance of several viral proteins. Overall, 82 viral proteins were quantified, including 77 canonical proteins and 5 novel open reading frames (ORF). Two, ORF1872 and US34, were up-regulated by both MG132 and bortezomib (**Figure 4**, **Table S5**), suggesting they were readily degraded *via* the proteasome during early infection. We previously identified ORF1872 as a putative unstructured and inherently unstable protein (Nightingale et al., 2018). Only glycoprotein gH (UL75) was up-regulated by MG132 in the absence of substantial upregulation by bortezomib (**Figure 4**, **Table S5**). gH was quantified in our previous multiplexed MG132/leupeptin analysis (Nightingale et al., 2018). Its rescue by MG132 and leupeptin but not bortezomib is likely to reflect lysosomal proteolysis after virion entry through endocytosis.

## Discussion

HCMV is known to be a master regulator of host immunity, achieving lifelong persistence in infected individuals by utilizing a wide range of strategies to modulate host protein expression. These include the deployment of proteins to target host factors for degradation. Here, we provide a searchable database that systematically details the route of degradation of cellular proteins during the establishment of a productive HCMV infection. Furthermore, this data can be used to predict molecules of key importance in antiviral immunity to HCMV on the basis of their degradation.

MG132 is a less selective proteasomal inhibitor than bortezomib, having previously been reported to inhibit lysosomal degradation pathways *via* inhibition of calpains and cathespsins (Kisselev and Goldberg, 2001), in addition to the proteasome. In our previous publication, 75% of proteins rescued by leupeptin at 12 h of infection were also rescued by MG132. The usefulness of comparing this broad proteasomal/lysosomal inhibitor with the specific proteasomal inhibitor bortezomib is the identification that 62–85% (34–47 proteins) of proteins rescued by MG132 were also rescued by bortezomib, suggesting that the proteasome is the predominant route for early protein degradation at 12 h post-HCMV infection. Overall, of all *downregulated* proteins, 44% were rescued by at least one of MG132 or bortezomib. It is possible that in order to downregulate certain proteins, HCMV must employ degradative pathways in order to achieve sufficiently rapid change in protein abundance.

We and others have previously shown that membrane proteins are targeted for lysosomal degradation during HCMV infection (Weekes et al., 2013; Fielding et al., 2014; Hsu et al., 2015; Fielding et al., 2017), and data here identified that all proteins rescued by MG132 but not bortezomib had a membrane origin. Certain proteins were exclusively degraded by a non-proteasomal route, including GLG1 and CHST14. Extension of these inhibitor studies to examining membrane-enriched samples, for example samples enriched for plasma membrane proteins (Weekes et al., 2013; Weekes et al., 2014) would therefore be of substantial interest, and may identify a distinct degradative route for proteins originating from these compartments.

Comparison of data from this study with our previous transcriptional analysis of host gene expression during infection at 24 hpi (Nightingale et al., 2018) suggested that 44 of the 81 proteins (54%) with MG132 and bortezomib rescue ratios <1.5 were more than 1.5-fold transcriptionally downregulated, which would be expected to be a major mechanism of protein downregulation in the absence of degradation (Tirosh et al., 2015). The fold change cut off of 1.5 for both downregulation by HCMV, and rescue by either inhibitor was based on a significance threshold of p <0.01, however had the effect of excluding proteins with “borderline” rescue ratios of >1.25 but <1.5. 39/81 proteins with MG132 and bortezomib rescue ratios <1.5 exhibited rescue ratios for MG132 or bortezomib or both that were nevertheless >1.25, suggesting that this group of proteins included some candidates that downregulated by degradation, at least in part.

Proteasome activity is necessary for efficient viral gene transcription and viral replication (Prösch et al., 2003; Kaspari et al., 2008; Tran et al., 2010b; Le-Trilling et al., 2016). More viral proteins were down- than up-regulated upon application of proteasome inhibitors at 12 hpi, including seven down-regulated >2-fold by both MG132 and bortezomib. One reason could be that cellular factors hindering viral gene transcription (e.g. ND10 components) are no longer degraded during HCMV infection in the presence of proteasome inhibition, leading to impaired expression of viral genes. This highlights that there are at least two mechanisms that could lead to the stabilization of a given host protein. The first is degradation of the host protein along the pathway inhibited by the drug. A second possibility is reduction by the drug of the abundance of a viral protein responsible for the degradation process. For instance, US22, which was down-regulated by MG132 and bortezomib, has been reported to function as an RNA-associated viral protein, thus has the potential to regulate gene expression post transcriptionally (Lenarcic et al., 2015). Although none of the viral proteins downregulated >2-fold by both MG132 and bortezomib or MG132 alone are known to target host proteins for degradation, their downregulation could potentially provide an alternative explanation for some of the changes we observed.

Overall, this analysis of host protein degradation during HCMV infection has not only identified proteasomal degradation as a key mechanism subverted by the virus early during infection, but has also generated a shortlist of proteasomally degraded proteins enriched in known HCMV restriction factors. Further investigation into the role of the other proteins in this shortlist is warranted to determine if they also have restrictive capabilities. Identification of HCMV restriction factors, understanding the mechanism by which they restrict infection and identification of viral antagonists that target these factors for degradation are of fundamental interest due to the potential for therapeutic intervention.

## Data Availability Statement

The datasets generated for this study can be found in the ProteomeXchange Consortium *via* the PRIDE partner repository (Perez-Riverol et al., 2019), dataset identifier PXD021961.

## Author Contributions

K-ML, KN, and MW designed the experiments. K-ML, KN, and MW wrote the manuscript. K-ML, KN, and RA performed the experiments. K-ML, KN, LS, and MW analyzed the proteomics data. K-ML, KN, LS, and MW edited the manuscript. MW supervised all research. All authors contributed to the article and approved the submitted version.

## Funding

This work was supported by a Wellcome Trust Senior Clinical Research Fellowship (108070/Z/15/Z) to MW and a strategic award to Cambridge Institute for Medical Research from the Wellcome Trust (100140).

## Conflict of Interest

The authors declare that the research was conducted in the absence of any commercial or financial relationships that could be construed as a potential conflict of interest.
