# Supplementary material for: Rapid Degradation Pathways of Host Proteins During HCMV Infection Revealed by Quantitative Proteomics
Source: Front Cell Infect Microbiol. 2021 Jan 27;10:578259. doi: 10.3389/fcimb.2020.578259 (PMC7873559; doi:10.3389/fcimb.2020.578259)
Supplement: Supplementary file 1 [file DataSheet_1.docx]

**Supplementary Materials**

**Supplementary table legends**

**Table S1. Details of each group of proteins shown in Figure 2B.** Each worksheet shows a different group of proteins illustrated in Figure 2B.

**Table S2. Interactive spreadsheet of all data in the manuscript.** The “Plotter” worksheet enables generation of graphs for all of the proteins quantified, and easy visualization of statistics. The “Data” worksheet shows minimally annotated protein and transcript data, with only formatting and normalization modifying the raw data. The “Stats” worksheet shows the p-values for each fold change or rescue ratio.

**Table S3. Details of TMT labelling used in this paper.**

**Table S4. Comparison the two MG132 screens.** Data related to **Figure S2**, comparing HCMV/Mock and (HCMV with 10 µM MG132)/HCMV from this manuscript and Nightingale et al 2018

**Table S5. Viral proteins dysregulated by proteasome inhibitors.** Lists of viral proteins that were up- or down-regulated > 2-fold by both proteasome inhibitors, as well as those that were dysregulated > 2-fold by one of the inhibitors but not the other.

**Supplementary figures and figure legends**

**
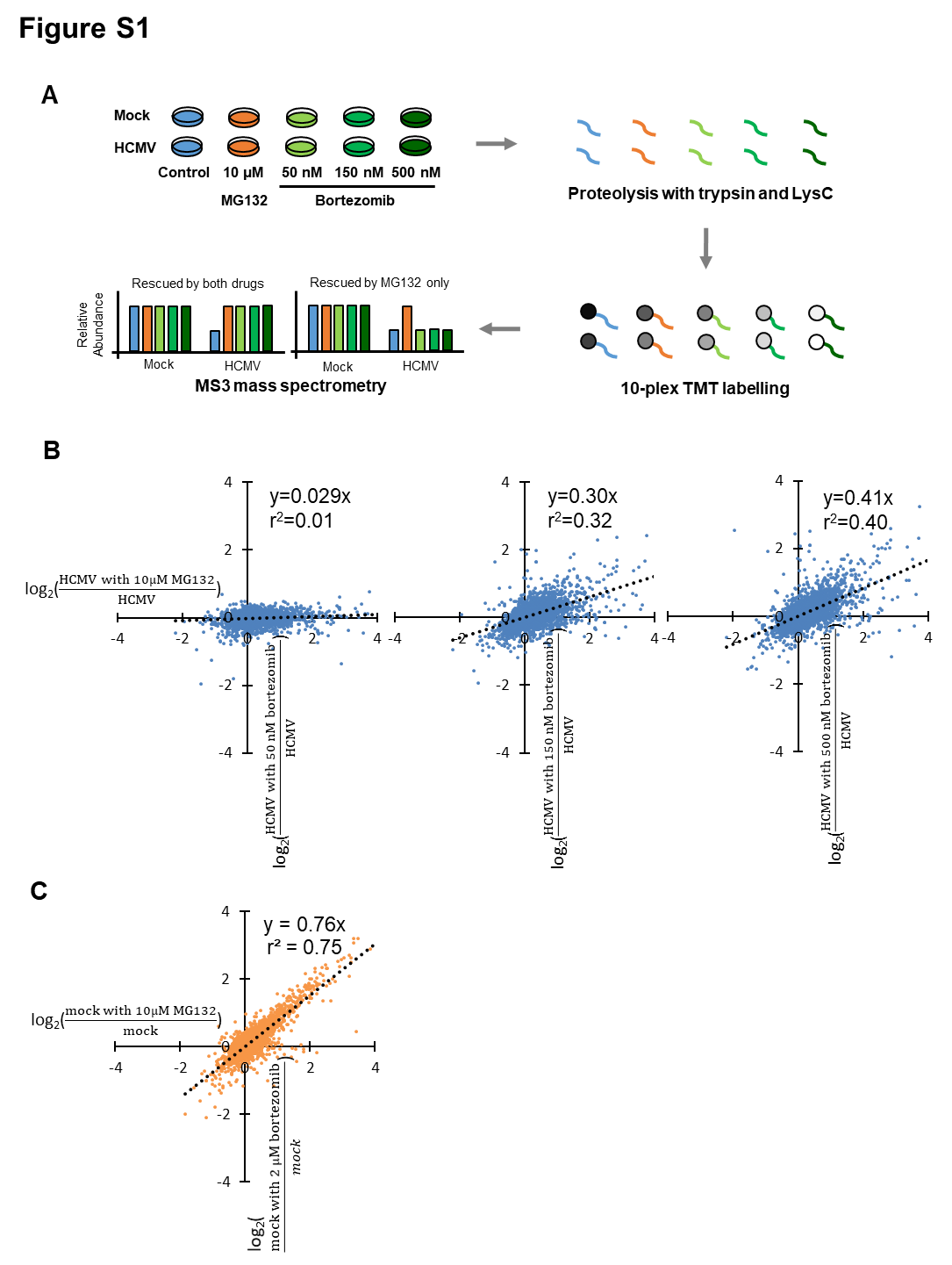
**

**Figure S1. Optimisation of bortezomib concentration by comparison with 10 µM MG132.** (A) Schematic of the experimental workflow. HFFF-TERT cells were infected with Merlin strain HCMV (MOI 5) or mock infected. After 12h of infection, cells were treated with 10 µM MG132 or 50 nM, 150 nM or 500 nM of bortezomib for a further 12 h and harvested for analysis at 24 hpi. Whole cell lysates were digested into peptides, which were labelled with TMT reagents followed by MS3 mass spectrometry. (B) Comparison of 10 µM MG132 with 50 nM, 150 nM or 500 nM bortezomib (bort). (C) Comparison of mock infection in the presence or absence of 10 µM MG132 with mock infection in the presence or absence of 2 µM bortezomib.


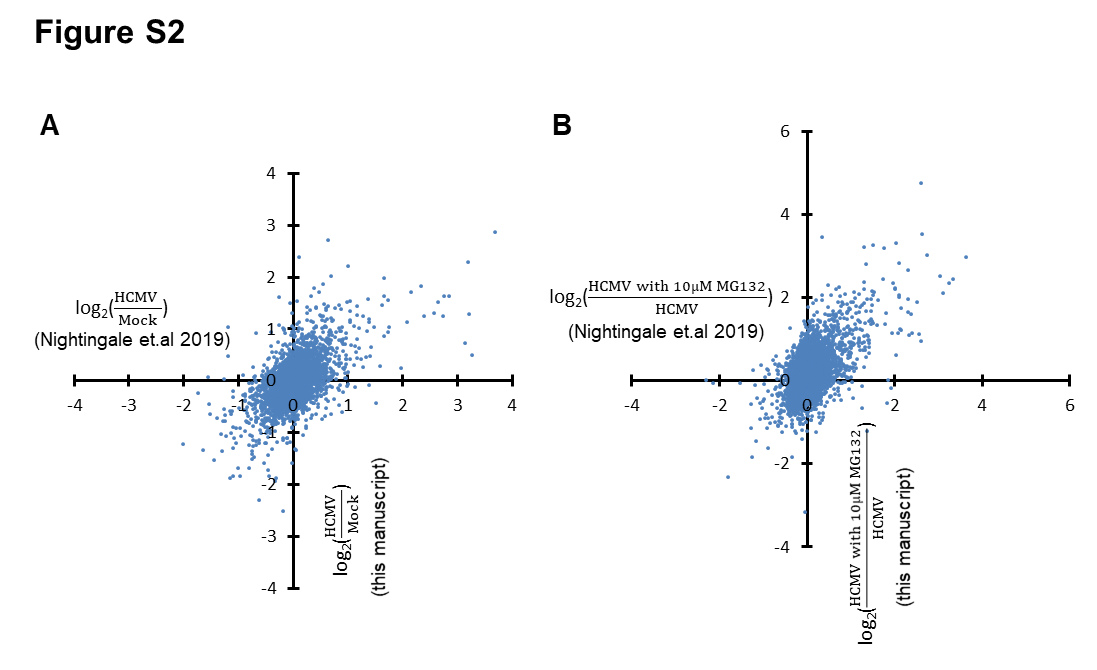


**Figure S2. Comparison of protein changes in the presence of MG132 at 12 hours of HCMV infection from this and our previous study**.


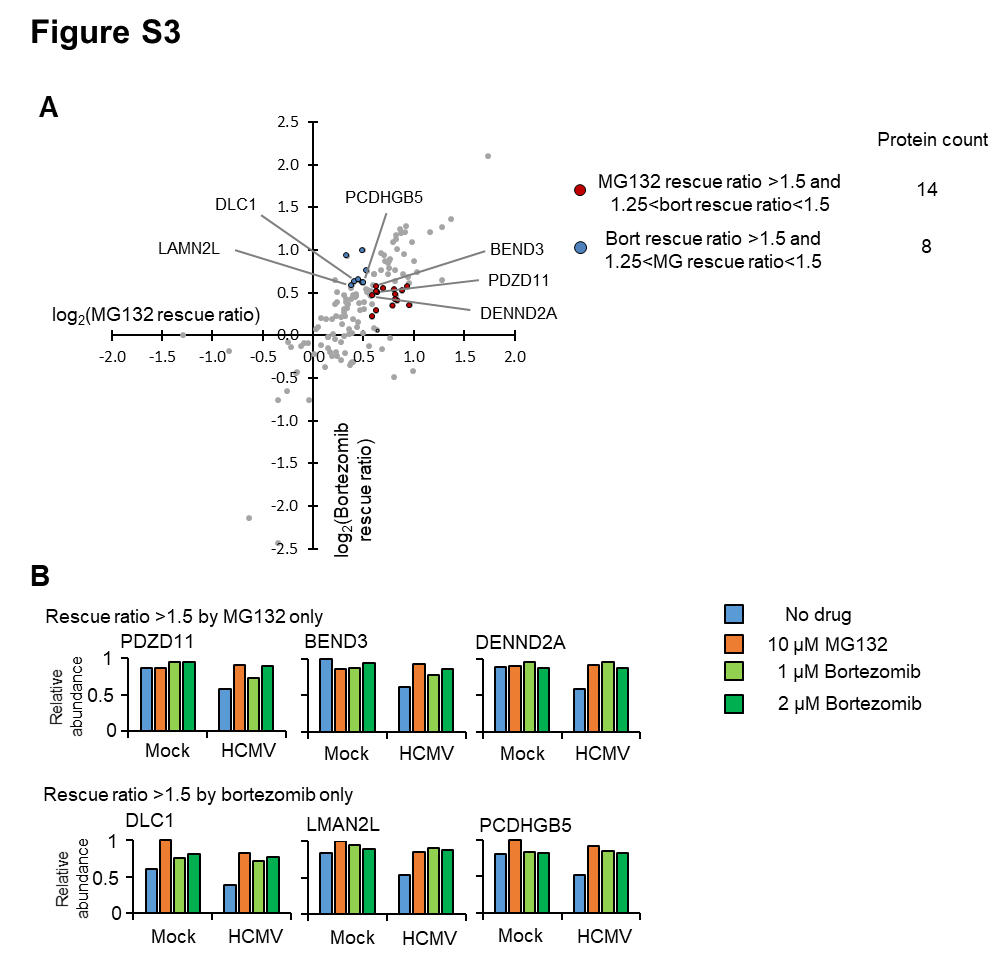


**Figure S3. Examples of proteins exhibiting rescue ratios >1.5 with only one of two inhibitors, but a rescue ratio between 1.25 – 1.5 fold with the other inhibitor.** (A) Results of the inhibitor-based screen. All 145 proteins downregulated >1.5 fold are plotted. Two groups were identified: proteins exhibiting rescue ratios >1.5 with MG132, but a rescue ratio between 1.25 – 1.5 fold with bortezomib (red), and proteins exhibiting rescue ratios >1.5 with bortezomib, but a rescue ratio between 1.25 – 1.5 fold with MG132. The table on the right shows the number of proteins in each group. (B) Examples from each group.

**Detailed materials and methods for proteomics analysis**

*Whole cell lysate protein digestion*

Cells were washed with PBS once, typrsinised, neutralised with complete DMEM, pelleted, and lysed (6M Guanidine [Thermo]/50 mM HEPES [Sigma] pH 8.5). Lysates were sonicated for 2.5 min at constant 4°C cooling with Bioruptor Pico (Diagenode), and cell debris was removed by centrifugation at 21,000 xg for 10 min at 4°C. To reduce protein, dithiothreitol (DTT, Sigma) was added and samples were incubated for 20 min at room temperature. Cysteines were alkylated with 14 mM iodoacetamide (IAA, Sigma), incubated 20 min at room temperature in the dark, and excess IAA was quenched with DTT for 15 min. Guanidine concentration was lowered to 1.5M with 200 mM HEPES (pH 8.5), then protein samples were digested with LysC protease (Wako) at a 1:100 protease-to-protein ratio for 3 h at room temperature. Guanidine concentration was further lowered to 0.5M and Trypsin (Thermo) was then added at a 1:100 protease-to-protein ratio followed by overnight incubation at 37 °C with constant shaking. Trypsin was quenched with 5% formic acid. Samples were then centrifuged at 21,000 xg for 10 min at 4°C to remove undigested protein. Peptides were subjected to octadecyl carbon chain (C18) solid-phase extraction (SPE, Sep-Pak, Waters) and dried with a speed-vac (Thermo).

*Peptide Labelling with Tandem Mass Tags*

Desalted peptides were dissolved in 200 mM HEPES (pH 8.5) and peptide concentration was measured by microBCA (Thermo). 2 μg of TMT reagents (dissolved in anhydrous acetonitrile [Acros organics]) was added to every μg of peptides at a final acetonitrile concentration of 30% (v/v) and incubated at room temperature for 1 h. The TMT-10 plex isobaric label reagent set (Thermo, Cat # 90110) was used for labelling. Sample labelling was as indicated in Table S3. The reaction was quenched with 0.3% (v/v) hydroxylamine (Thistle Scientific). Equal amounts of TMT-labelled samples were combined and subjected to C18 SPE then dried by speed-vac before being subjected to high pH reversed phase fractionation (HpRP) or mass spectrometry.

*Offline High pH Reversed-Phase Fractionation*

TMT-labelled peptides were fractionated using an Ultimate 3000 rapid separation (RS) nano UHPLC system (Thermo), generating 12 combined fractions. The system is equipped with a Kinetex Evo C18 column (Phenomenex) that has 2.1 mm in internal diameter (ID) and is 25 cm in length, filled with C18 bound silica particles with diameter of 1.7 μm. Mobile phase was HPLC grade H_2_O, acetonitrile, and ammonium formate (pH 10). The concentration of ammonium formate was maintained at 20 mM while concentration of acetonitrile gradually increased throughout the fractionation elution programme. Starting from 2.7% (v/v), acetonitrile concentration increased to 21% in the first 10 min, to 36% after 24 min 15 sec of elution, then to 51% after 33 min of elution. Acetonitrile concentration was subsequently increased and maintained at 90% for 10 min to wash the column. The flow rate was 400 ml/min and the elution was performed at 45°C. UV absorbance was monitored at 280 nm and fractions were collected into 96 well microplates using the integrated fraction collector. Fractions were recombined orthogonally in a checkerboard fashion, combining alternate wells from each column of the plate into a single fraction, and commencing combination of adjacent fractions in alternating rows. Wells were excluded prior to the start or after the cessation of elution of peptide-rich fractions, as identified from the UV trace. This resulted into two sets of 12 combined fractions, which were dried in a vacuum centrifuge and resuspended in 10 ml solvent (4% acetonitrile and 5% formic acid) prior to LC-MS3.

*Liquid chromatography coupled with multi-stage mass spectrometry (LC-MS3)*

An Ultimate 3000 RSLC nano UHPLC was used for online fractionation, equipped with a 300 μm ID x 5 mm Acclaim PepMap μ-Precolumn (Thermo) and a 75 μm ID x 50 cm 2.1 μm particle Acclaim PepMap RSLC analytical column (Thermo). Loading solvent was 0.1% formic acid. Analytical solvent contained HPLC grade H_2_O, acetonitrile, and formic acid. Samples were loaded at 5 ml/min for 5 min in loading solvent before beginning the analytical gradient. Formic acid concentration was maintained at 0.1% during the analytical gradient while concentration of acetonitrile gradually increased. All separations were carried out at 55 °C. Mass spectrometry data were acquired using Orbitrap Lumos mass spectrometer (Thermo). TMT-based analysis used a MultiNotch MS3-based method (McAlister et al., 2014). MS1 scans surveyed 380-1500 Th, with resolution of 120,000, automatic gain control (AGC) target of 2 x 10^5^, and maximum injection time of 50 ms. Ions that had the counts of 5 x 10^3^ counts and above triggered MS2 analysis, with Quadrupole isolation at an isolation width of 0.7 Th, normalised collision energy (NCE) set to 35% for CID fragmentation, 1.5x10^4^ AGC target, and 120 ms maximum injection time. Top 6 MS2 ions were selected for HCD fragmentation (NCE 65%) in MS3. MS3 resolution was 60,000, with an AGC target of 1 x 10^5^ and a maximum accumulation time of 150 ms. The entire MS/MS/MS cycle had a target time of 3 sec. Dynamic exclusion was set to +/- 10 ppm for 70 sec.

*Protein Quantification*

Mass spectra were processed using “MassPike”, which is a SEQUEST-based software for quantitative proteomics, developed by Professor Steven Gygi and colleagues at Harvard Medical School. In MassPike, MS spectra were converted to mzXML format using an extractor built upon Thermo Fisher’s RAW File Reader library (version 4.0.26). The standard mzXML format has been augmented during extraction and conversion, with additional customisations that are specific to ion trap and Orbitrap mass spectrometry and essential for TMT quantitation. These customisations consider ion injection times for each scan, Fourier Transform-derived baseline and noise values calculated for every Orbitrap scan, isolation widths for each scan type, scan event numbers, and elapsed scan times.

Mass spectra acquired were searched against a combined protein sequence database that includes human proteins, HCMV proteins, and possible protein contaminants that might be introduced to samples. The human protein Uniprot database was downloaded on 26^th^ January, 2017. An HCMV protein database was assembled from the HCMV strain Merlin Uniprot database, non-canonical human cytomegalovirus ORFs described by Stern-Ginossar et al (23180859), and a six-frame translation of HCMV strain Merlin filtered to include all potential ORFs of ≥8 residues (delimited by stop-stop rather than requiring ATG-stop). The database also included common contaminants (bovine serum albumin and porcine trypsin, and annotated human protein contaminants such as keratins). Searches were performed using a 20 ppm precursor ion tolerance. Fragment ion tolerance was set to 1.0 Th.

TMT tags on lysine residues and peptide N termini (229.162932 Da) and carbamidomethylation of cysteine residues (57.02146 Da) were set as static modifications, while oxidation of methionine residues (15.99492 Da) was set as a variable modification.

Peptide identification was executed in the order of the ranks using cross-correlation score (XCorr), as the correctness of peptide spectral matches (PSMs) decreased along the ranks. A target-decoy strategy was employed to ensure the quality of peptide identification (Elias and Gygi, 2007). A decoy database was generated by reversing the sequence of the composite protein database detailed above. Assignment of peptides from this decoy database were considered as a “false discovery”, and peptide identification terminated before the false discovery rate reached 1%. Correct and incorrect spectral matches were distinguished from one another using linear discriminant analysis based on several different parameters including XCorr, the XCorr difference between top and second possible peptide (ΔCn), precursor mass error, and charge state.

Protein assembly was performed by principles of parsimony to produce the smallest set of proteins necessary to account for all observed peptides, meaning in cases of redundancy, shared peptides were assigned to the protein sequence with the greatest number of matching unique peptides.

Following fragmentation, each TMT tag produces reporter ions with specific mass, which were surveyed in low m/z area of the MS3 spectrum. The maximum intensity nearest to the theoretical m/z of each reporter ion was used. Proteins were quantified by summing TMT reporter ion counts across all matching peptide-spectral matches. If a TMT experiment uses n (number) types of TMT tags, more than n-1 TMT channels missing and/or a combined signal-to noise ratio of less than 25n across all TMT reporter ions were considered poor quality of MS3 spectra. PSMs with poor or no MS3 spectra were excluded from quantitation. Protein quantitation values were exported for further analysis in Excel. The method of significance A was used to estimate the p-value that each ratio was significantly different to 1 using Perseus version 1.5.1.6. Values were adjusted for multiple hypothesis testing using the method of Benjamini-Hochberg (Cox and Mann, 2008).

**References related to detailed materials and methods for proteomics analysis**

Cox, J., and Mann, M. (2008). MaxQuant enables high peptide identification rates, individualized p.p.b.-range mass accuracies and proteome-wide protein quantification. *Nat Biotechnol* 26**,** 1367-1372.

Elias, J.E., and Gygi, S.P. (2007). Target-decoy search strategy for increased confidence in large-scale protein identifications by mass spectrometry. *Nat Methods* 4**,** 207-214.

Mcalister, G.C., Nusinow, D.P., Jedrychowski, M.P., Wühr, M., Huttlin, E.L., Erickson, B.K., Rad, R., Haas, W., and Gygi, S.P. (2014). MultiNotch MS3 enables accurate, sensitive, and multiplexed detection of differential expression across cancer cell line proteomes. *Anal Chem* 86**,** 7150-7158.
